# Participation in Church or Religious Groups and Its Association with Health. Part 2: A Qualitative, Canadian Study

**DOI:** 10.1007/s10943-014-9961-9

**Published:** 2014-11-11

**Authors:** Valerie Michaelson, William Pickett, Peter Robinson, Linda Cameron

**Affiliations:** 1 School of Religion, Theology Hall, Queen’s University, Kingston, ON l7L 2W1 Canada; 2 Department of Public Health Sciences, Queen’s University, Kingston, ON Canada; 3 Wycliffe College, University of Toronto, Toronto, ON Canada; 4 OISE, University of Toronto, Toronto, ON Canada

**Keywords:** Adolescence, Church involvement, Emotional health, Forgiveness, Health

## Abstract

As part of a mixed-methods study, this qualitative inquiry determined how adolescent participation in church or religious groups related to their health. We used grounded theory with a phenomenological approach to inquiry. Consistent with the quantitative findings, children (*n* = 12) involved in religious groups reported lower participation in risk behaviors, higher pro-social behaviors, but poorer levels of emotional well-being and physical health. Findings raise theological and practical questions about the practices and teaching of the church with respect to children’s ministry. They suggest an emphasis on teaching about behaviors and morality rather than a more integrative message involving the whole of life.

## Introduction

Church involvement, as a context within which a person learns to live in relationship with God, humanity and the world, has the potential to positively transform the lives of young people. At its best, the message of the church is one that involves every part of life; it is alive with hope and expectation. Core to this message is the incarnation of Jesus, which signals the full embodiment of the life that God intends for humans and God’s commitment to realize that life. Experiences of redemption and love offered in the gospel are ones that reach into every part of existence. If churches are grounded in this message, both in theology and practice, then one would expect to see that regular participation in church would be protective in terms of the health of young people. Tangible effects would include better indicators of emotional and physical health and reduced engagement in behaviors that compromise health.

In a recent national quantitative study, we had a unique opportunity to examine participation of young Canadians in church and other religious groups, as well as relations between such participation and a large variety of health indicators (Michaelson et al. 2014). Our findings suggested that while regular church involvement is protective to the health of young people in terms of reported engagement in overt risk behaviors (most evident for substance use and sexual behaviors) as well as pro-social choices, this protective trend disappeared for indicators of emotional health and well-being. Our quantitative findings were novel and provocative and raised many theological and practical questions for church ministry. This in turn led to a further qualitative inquiry, reported here. The purpose of this qualitative study was to obtain deeper insights from young people surrounding the potential influence of church involvement on their health and health behaviors. In doing so, we obtained a richer understanding of the relationships suggested by the quantitative component of our study. Combined, our hope was our national quantitative findings, and our in-depth but more regional qualitative findings would provide deep and rich insights into this very practical area of theology. Ultimately, our hope was that we would provide new evidence to help young people thrive in an increasingly complex world.

## The Potential of Church Ministry

Church ministry is supported by a theological framework that encompasses the whole of life. Jesus invites us into the fullness of life; *a new way of being* in the world. While our behaviors reflect this call, good behavior is not an end in itself. It is a response to or a result of a relationship with a dynamic and living God (Torrance 1996; Wright 2008).

Broadly speaking, while the church exists within a fallen and broken world and is itself marred by brokenness, the church still remains central to God’s bringing about the fullness of life in Christ in this world. God uses the church to draw us into participation in a renewed life with God, one another, and creation. Based upon this theological premise, in our program of research with young people, we expected to find that involvement in a church community would lead to improved reports of health and well-being. Children today are growing up in an increasingly complicated world, and it is thus all the more important that in the church, we are intentional in nurturing them, and that they are provided with tools to navigate their lives faithfully and well, and supported in healthy growth and development.

## Our Qualitative Study

The goal of the qualitative component of our mixed-methods study was to develop a deeper understanding of the human experiences that underlay our quantitative findings, beyond speculation. More specifically, we hoped that a qualitative inquiry would provide novel insight into the experiences that some young people were having in the Canadian church, and its potential influence on many aspects of health.

### Methodology

This qualitative inquiry aimed to provide a deeper examination of the major patterns observed in the previously published quantitative findings. Our objective was to provide evidence in support of a theory about relationships between church involvement and child health. There was a phenomenological component to this study in which we were interested in the participants’ understandings of their own lived experience of church involvement; however, our chosen methodology was grounded theory (Charmaz 2006). The study protocol was approved by the University of Toronto Social and Behavioural Research Ethics Board (Protocol Reference 26986).

## Procedures

### Overview

Detailed in-person interviews were conducted, and open-ended questions and prompts were used to elicit a multi-textured understanding of the study phenomena. These interviews facilitated extensive discussions about each participant’s experiences of church and its potential effects on their health. Through these exchanges, we gained an increasingly more focused and specific level of observation.

Our initial questions aimed to explore issues stemming from the quantitative findings. These focused on: choices to participate in overt risk behaviors, similar choices to participate in pro-social behaviors, experiences with physical health, and experiences with emotional health (specifically, feelings of loneliness, helplessness, “wishing I was someone else” and regret). Later questions explored how each participant’s actual experiences connected with Jesus’ invitation into the fullness of life.

### Sample

The qualitative sample consisted of 12 church-involved children. Church leaders were asked to invite participation from young people in their congregation who had a meaningful engagement with their church community. Diversity of sample was sought by way of age (11–15), sex, schooling (public, Roman Catholic, Christian and home–school) and denomination (Anglican, Baptist, Christian Reformed, Free Methodist and Roman Catholic). All recruits self-identified as “being involved in their church community.” Further, all 12 reported positive family relationships and also positive relationships with their respective churches. This interview sample was therefore highly selective and included an unusually stable core of children. The potential positive bias in study results was recognized interpretively.

### Data Collection, Coding and Analysis

Data collection involved in-depth interviews in which participants were invited to share their experiences of church involvement in relation to specific health indicators and relations. Central to grounded theory is simultaneous involvement in both data collection and analysis. Thus, data were analyzed and coded throughout the data-collection process, and the theory and associated interview questions became increasingly more focused and refined as the study progressed.

In order to identify each participant’s emergent descriptions of thought patterns, feelings and actions, interviews were transcribed verbatim and consecutively examined line-by-line. Data were then coded, and emergent themes were identified and compared to verify their descriptive content and to confirm that themes were grounded in the data. As a second stage, the codes were sorted into categories through a constant comparison process. Categories were then organized, and data collected at later stages in the study were used to add, elaborate, and saturate codes and categories and to identify core phenomena. In practice, the steps of analysis were not strictly sequential. Consistent with contemporary grounded theory practice, analyses were done iteratively, moving forward and backward, constantly collecting data and reexamining data, codes, categories and the whole model (Charmaz 2006).

## Primary Findings

**Fig. 1:**
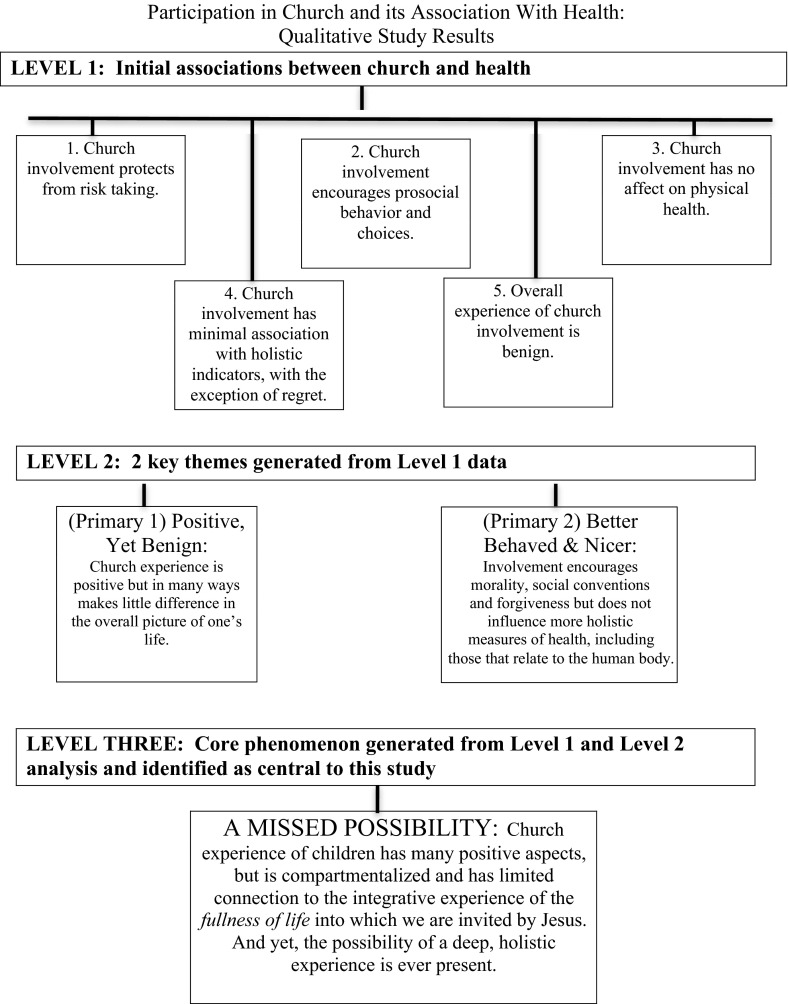
Qualitative study summary

Overall, findings suggest that while the church experience of children has many positive aspects, it also suggests that children are experiencing a compartmentalized faith that has limited or no connection to the fullness of what the Christian life can be. These results are not abstract findings that are disconnected from real lives. Rather, they are embedded in real stories of young people who shared their own journeys of faith and church. Themes were organized into three levels, and a model that summarizes these recurrent themes is presented in Fig. 1. Level-one findings related to different components of health: risk behaviors, pro-social behaviors, physical health and emotional well-being. In level two analyses, two broad themes emerged: (1) *church involvement is positive, yet fairly benign in the lives of these children* and (2) *church involvement encourages positive moral behavior, pro-social choices, forgiveness, and* (*to a lesser degree*) *positive feelings about oneself, but has little to do with the human body or measures of emotional well*-*being.* Through a process of theoretical sampling, one overarching theme—or core phenomena—was identified as central to this study: *the church experience of participants is characterized by a positive* (*yet fairly benign*)*, compartmentalized and moral behavior*-*based experience of the Christian faith.* However, enough positive aspects of participants’ church life were present that the possibility of a vital, integrative faith always seemed to be possible. We therefore described this scenario as “a missed possibility.”

## Level 1 Reporting: Initial Associations Between Church and Health

A summary of Level 1 observations and associated emergent themes is presented below. Interviewer questions and comments are in regular font, and the participants’ responses and comments are in italics and a bolded font.

All participants agreed that their church experiences impacted their decisions to engage or not engage in overt risk-taking behaviors that might compromise their health. When asked for examples, participants cited: “not lying,” “not doing drugs,” “not drinking,” “not stealing,” “not playing video games 24/7,” as well as “what I do with my friends” and “how I should act.” In the words of one participant, **“[** ***my church*** **]** ***helps me, um, know if I should do something or not*** **”** (participant 10). Participants were positive about the church’s role in their lives and consistently shared a view that church involvement both helps them to resist participation in overt risk behaviors and to set their own moral compasses.

Findings surrounding relations between church involvement and pro-social behavioral choices were revealing. When participants were asked “Does being a Christian make you nicer?” all but two replied with a definitive “yes.” (The remaining two participants replied “maybe.”) When asked why this might be, participants responded: **“** ***Because as a Christian we*** **’** ***re supposed to like love our enemy and treat someone as you want to be treated*** **”** (interview 9) as well as **“[** ***Going to church*** **]** ***makes you a better person*** **”** (interview 4).

In general, when participants talked about being healthy, consistent with medical and public health model orientations, they reported aspects of health connected to risks for illness and health promotion. For example, health is: **“** ***if you*** **’** ***re not sick*** **”** (interview 5**)** and **“** ***if you are active and you eat well*** **”** (interview 8). Another described a healthy person as *“* ***Someone who cares about their body, you take care of yourself… like just basically like exercise, just doing things to make sure your body stays strong and healthy*** **”** (interview 9).

For most participants, their understanding of good health did not extend beyond the aspects of health connected to the physical. On being asked: “What influence do you think church involvement has on health?” One participant responded: **“** ***I don*** **’** ***t really know. As far as all of the things I thought of church affecting, I don*** **’** ***t think health has been one*** **”** (interview 2). A pronounced theme was beginning to emerge: the relationship between church involvement and one’s body is very much connected to abstaining from morality-based risk behaviors and has little to do with physical health. This was emphasized in a comment from this next participant who was asked: What do you think health is?

**“** ***Umm….. uhhhhh…. health… I guess the shape of your body, the condition of it, not shape.*** **”**

Does the church ever say anything about the body? Taking care of your body?

**“** ***Ya, they say that doing things to hurt your body is bad… like drugs, alcohol…*** **”**

(interview 2).

The overarching theme that emerged is consistent with one reported in our quantitative study: while religiously connected children reported less health compromising risk behaviors, this trend disappeared when measured in terms of physical health.

Findings surrounding measures of emotional well-being were also telling. Our quantitative results suggested that while church involvement has positive associations with reduced engagement in risk behaviors and higher tendencies toward pro-social choices, it has little impact on indicators of emotional health. This was reflected in reported relations between church involvement and feelings of loneliness, helplessness, “wishing I was someone else” and regret.

With respect to loneliness, participants communicated a general sense that church involvement had little to do with its alleviation and that family, friends and extracurricular involvement had much more positive impacts. When we specifically asked “Do you feel lonely?” only half the participants affirmed regularly experiencing this feeling. One participant was very specific in her explanation that while she does not feel lonely, her church involvement is not connected to the reasons why she does not feel lonely (interview 5). In all participants, little connection was reported between feelings of loneliness (positive or negative) and church involvement.

Most participants also did not associate church involvement with decreased feelings of helplessness. The young people were asked if their experience of church helped to handle problems in the world or in life. A range of answers was reported: **“** ***A bit, ya*** **”** (interview 4); **“** ***Umm… maybe… I don*** **’** ***t know…*** **”** (interview 5); **“No, not really”** (interview 6) and **“** ***No*** **”** (interview 7). For the most part, there was not a strong sense that church involvement was helpful in terms of their abilities to handle problems. While God, church and (perhaps most strongly) prayer were sometimes connected with handling problems, or with decreased feelings of helplessness, no strong theme or pattern emerged. Some felt helpless; some did not. Some felt church helped them to handle things in the world; most did not.

Reports surrounding the possible influence of church involvement on feelings of “wishing I was someone else” were also somewhat modest. We began by asking participants: “Do you feel accepted and valued for who you are?” Most responded positively, and one participant emphasized that it is her faith in God that helps her feel more comfortable in who she is (interview 9).

When participants were asked: “Do you ever wish you were someone else?” most reported “no.” One participant said: **“** ***Sometimes… but then I am glad I am myself again*** **”** (interview 10) and another said: **“** ***If I wasn*** **’** ***t a Christian I probably wouldn*** **’** ***t feel that good about myself*** **”** (interview 4). Another participant made an immediate connection between feelings about themselves and their faith:

**“** ***Each person was created in their own way for God*** **’** ***s purposes…. If I don*** **’** ***t want to be changed based on what I know of what*** **’** ***s happening in my life so far, which is we are all in the Lord*** **’** ***s image… then I won*** **’** ***t be changed that way. That*** **’** ***s the way I see it*** **”** (interview 1).

Overall, while the relationship was modest, church involvement seemed to positively impact participants’ senses of self-identity and of feeling loved and accepted for who they are.

Of the initial three emotional health indicators (feelings of loneliness, helplessness and “wishing I was someone else”), only the latter appeared to be positively related to church involvement. This was surprising as it ran counter to the quantitative findings. Indeed, the fact that only one of the twelve children interviewed reported “wishing to be someone else” suggests that for these participants, church involvement may play a role in their positive sense of identity. This discrepancy could potentially be explained by the highly selective nature of our sample of young people. These interviews also suggest that for some children, church involvement may have a protective relation with a positive sense of identity.

Findings surrounding associations between church involvement and feelings of both loneliness and helplessness were more in keeping with our quantitative findings: that a meaningful church connection did not appear to provide a protective effect to children in the areas of decreased feelings of loneliness or decreased feelings of helplessness. That said, throughout the interviews, there were some glimpses of experiences suggesting that church involvement can indeed offer a protective influence and that the potential of the church to foster emotional well-being is ever present. The current normative experience, however, is that it does not.

The final indicator of health from our quantitative study that was of interest qualitatively was regret, specifically worded: “Do you feel sorry for things you do?” This measure had been an anomaly in the initial quantitative study. It was actually unclear how survey respondents had understood and interpreted the question itself. In one sense, this question could be understood as having overwhelming and/or debilitating feelings of guilt—a negative health indicator. However, if feelings of being “sorry for things you have done” (or as qualitative participants later interpreted this, having “feelings of regret”) were connected with both taking responsibility for one’s actions and with a deep experience of forgiveness, it could be viewed as a positive effect. It was our hope that these qualitative interviews would provide insight and a deeper understanding of this phenomenon.

Our strategy to understand this measure was to first explore participants’ experiences of forgiveness. They reported: **“[** ***Forgiveness*** **]** ***is when someone does something wrong and they say like I*** **’** ***m sorry and you forgive them and then you are friends again and you forget about it*** **”** (interview 5). Further: **“** ***It means saying sorry and forgetting about it, placing it in the past*** **”** (interview 10). For others, responses were more specific to Christianity:

**“** ***When I think of forgiveness, I think of two different things. Like if someone does something mean to you and you forgive them and they say sorry to you and kind of like it*** **’** ***s ok that you did that. I might have felt hurt inside about that but you*** **’** ***re saying sorry and you*** **’** ***re saying you didn*** **’** ***t mean to say those things, but when I think of another forgiveness I think of like God forgiving all your sins, like Jesus taking your sins on the cross*** **”** (interview 12).

Throughout the interviews, forgiveness was the theme participants talked about most readily, most passionately and with the most theological depth. They also spoke about forgiveness at greatest length and had the easiest time connecting it to their everyday as well as to their church experiences.

Participants unanimously agreed that regret was a good thing that helped one move from a wrong doing to forgiveness. Here are some representative responses: **“** ***Well, like if I do something bad it*** **’** ***s good to feel sorry.*** **”** Why? **“** ***Cause then if you didn*** **’** ***t feel sorry at all and you were glad you had hurt them or something it wouldn*** **’** ***t be nice*** **”** (interview 5); **“** ***Probably*** **[** ***feeling regret is*** **]** ***a good thing because if you feel bad for it then it means you inside know the right thing to do*** **”** (interview 2); **“** ***I think*** **[** ***regret*** **]** ***is a good thing…. If you didn*** **’** ***t*** **[** ***have regret*** **]** ***, you*** **’** ***d do stuff and not care about it*** **”** (interview 4) and **“** ***I suppose*** **[** ***feeling regret*** **]** ***helps you not do it again*** **”** (interview 7).

Participants consistently understood regret as an important element of forgiveness: **“** ***You have to feel sorry about it to receive forgiveness and apologize, or else it*** **’** ***s just fake*** **”** (interview 1) *and further:* **“[** ***If you didn*** **’** ***t feel sorry*** **]** ***it wouldn*** **’** ***t be a good thing because then you wouldn*** **’** ***t realize what you are doing is wrong and… you wouldn*** **’** ***t like… like when you*** **’** ***ve committed sin you know… but if you never have that feeling then you won*** **’** ***t always know when like you need to ask for forgiveness*** **”** (interview 9). Though older participants were much more nuanced in their responses, there was considerable agreement that regret is necessary for forgiveness to be meaningful and that it is a positive quality in their lives.

Not only did participants understand forgiveness as important, they saw it as both central to their church and home experience and as something that distinguished them from their non-church-connected friends and peers. For example:

Do you think [forgiveness is] something you understand more deeply because of your church connection?

**“** ***I wouldn*** **’** ***t understand any of this without my church connection.*** **”**

So how do the other kids at school do it?

**“** ***They don*** **’** ***t really.*** **”**

Can you take a guess what it’s like for them?

**“** ***Ya…. it*** **’** ***s like…. fights…. Someone is doing something mean to someone else, then after someone gets back at them, and then so on and so forth, then after both of them end up sitting in the principal*** **’** ***s office to get dealt with*** **”** (interview 1).

The strength and consistency of the responses suggest that the religiously connected participants who took part in the quantitative (health survey) component of the mixed-methods study understood “feeling sorry for things I do” as a positive health indicator connected with both taking responsibility for one’s actions and as a precursor to forgiveness. If our qualitative findings are generalizable to the larger, quantitative sample, it would suggest that church involvement offers a protective health effect, indicating that this group of young people is taking responsibility for their own actions and that regret is a precursor to a meaningful experience of forgiveness.

When participants were asked about their overall experience of church, for the most part, they were positive. All participants reported at least something good about their church, and most reported liking their church very much. Participants made observations such as: **“** ***It gives me comfort*** **”** (interview 1) and **“[** ***There is a*** **]** ***friendly attitude*** **”** (interview 2). One participant reported that he would really miss church if he did not have it (interview 10), another agreed that her church was really good (interview 7), and many participants reported a sense of belonging at church. It was clear from most participants that not only did they like their respective churches, but that for the most part, they perceived their own experiences at church to be positive.

Participants were, however, less certain that churches had meaningful impacts on their lives. For example, when asked “Does church have a big impact on your life?” a characteristic response was **“** ***I don*** **’** ***t really think so*** **”** (interview 6). When asked what difference her church connection makes in her life, another responded: **“** ***….. probably not a huge one*** **”** (interview 5). As soon as she said this, however, she reaffirmed: **“** ***But I really like*** **[** ***my church*** **]”** (interview 5). Early on in data collection, we observed the emergence of a theme that would become pivotal: while the church experience of most participants was positive, most also were clear that overall, their church experience was not (nor should it be) relevant to the rest of their lives. We have called this phenomenon: “positive, yet benign.”

## Level 2 Reporting: Associations Between Church Involvement and Health: Emergent Themes

Throughout the observation, coding and analysis of the level-one findings, two main themes emerged:

1. The church experience of participants, while having many positive aspects, was perceived as benign (it made limited difference) in the overall lives of participants.
2. While associations between church involvement and health emerged throughout the study, they included an emphasis on moral behavior, social conventions, forgiveness and a possible relationship with positive identity. Church involvement did not appear to have associations with either physical health or with other emotional measures of health.

These emergent themes suggest that the church experience of participants is facilitating a disintegrative experience of the Christian life that has a disproportionate emphasis on moral behaviors, at the expense of having a more integrative experience of life. While the church experience was not necessarily negative, our qualitative findings suggested that young people were potentially missing out on much of the richness and depth that the Christian life has to offer.

At this stage, the interview questions were re-focused in order to encourage participants to articulate ways that they saw their church experience connecting with bigger parts of their lives: with their interests, school and the things that bring them joy:

Do you think church is involved in a part of your life or the whole of your life?

**“** ***I think the whole of my life.*** **”**

So would church make a difference when you play hockey? (Note to reader: ice hockey is a very common activity among young people in Canada.)

**“** ***Well, not really 100*** ***%, but I*** **’** ***d say maybe 99*** ***%. Well I guess with hockey because I*** **’** ***m not mouthing off to my coach and stuff*** **”** (interview 2).

What is interesting in this participant’s response is that even though she had previously talked at great length about her joy in playing hockey, and the innate physicality of that sport, when asked how it connected to her church experiences, she pointed immediately back to an issue of behavior.

The story that was emerging is certainly not all negative. Indeed, it is appropriate and positive that the church is intentional in its teaching about avoiding common risk behaviors that can be destructive to one’s overall health (emotional, physical, mental and spiritual). There are many positive aspects to the church experience of these participants, including a deep experience of forgiveness. The challenge here was not that church involvement seems to offer a protective effect in areas of risk behaviors and pro-social behaviors, but that participants generally do not see that church involvement is influencing other areas of life. As this pattern continued to emerge throughout many conversations, we observed a consistency in reports of a compartmentalized and behavior-oriented experience both of church involvement and of Christianity.

## Level 3 Reporting: Associations Between Church Involvement and Jesus’ Invitation into the Fullness of Life

One core phenomenon emerged at the heart of this study: **while the church experience of children has many positive aspects, it is a compartmentalized faith that has limited connection to most areas of life.** In other words, these children are in many ways not benefiting from the richness and depth of the Christian life that can be offered through the Church. And the Christian faith, inherently, is about life.

In these latter interviews, participants were asked to imagine what the “the most wonderful, fullest possible life that Jesus has for us in this world” would look like. One participant said this:

**“** ***I*** **’** ***d do everything perfect.*** **”**

So it would have to do with your behavior?

**“** ***Ya. Because sometimes I tend to do stuff I probably shouldn*** **’** ***t.*** **[** ***The fullness of life*** **]** ***would be totally perfect. I mean, it*** **’** ***s just human to do stuff that is wrong… it would be very strange*** **”** (interview 6).

For this participant, perceptions surrounding the life that Jesus wants for her were clearly connected with behavior.

Probes were then used to encourage participants to share something more deeply connected to a life-giving relationship with God:

Would [the fullness of life] also have to do with how much fun you have or how much joy you have?

**“Ya. How much fun I have.”**

So it would also be connected with fun?

**“Well…. being totally perfect… would probably influence how much. I’m not really sure but it might influence how other people want to be around you. It might make some people not want to be around you.”**

So you might have fewer friends?

**“I’m guessing”** (interview 6).

This comment was surprising as this participant had earlier shared a very positive picture of his church and of being a Christian. And yet, when pushed to really think about what this fullness of life might look like, in the same breath as he connected it with fun he also thought that it might mean that he had fewer friends. We continued to explore this participant’s thoughts and perceptions:

So does that kind of life [that Jesus wants for you] sound good, or not so good?

**“** ***Not so good.*** **”**

I just want to make sure I’m understanding you. If you lived the way Jesus wanted you to all the time… you’d be perfect and there would be good things in that but some people might not want to be around you so it wouldn’t be that good?

**“** ***Well, it might not be.. The people might.. might want to be around you but…***

***Maybe I want to hit someone…I*** **’** ***d be so angry and then I couldn*** **’** ***t do that if I was mad at them.*** **”**

So you’d have to hide your mad feelings?

**“** ***Ya. That*** **’** ***s hard. I have really big temper.*** **”**

So would you not be able to be who you are?

**“** ***Ya.*** **”**

So to live in this perfect way you wouldn’t really be able to be fully yourself?

**“** ***Yes*** **”** (interview 6).

For this participant, living fully in Jesus implied that he would be unable to be himself. This was telling. While this participant had readily made connections between church involvement and forgiveness and between church involvement and choices to not engage in risk behaviors, he saw very little connection between church involvement and the larger picture of his life. This response was in keeping with much of what had been reported throughout the interviews and appeared to be sadly missing the mark on the potential the church has to live out the depth of its own story and draw children into life in Christ. We identified this core theme as: *A Missed Possibility*: *While the church experience of children has many positive aspects, it is compartmentalized and has limited connection to the integrative fullness of life into which we are invited by Jesus.*

One obvious concern raised by this qualitative study is that church involvement has the potential to be so much more. It has the potential to nurture God’s children in holistic experiences of overall health that extend into every part of their lives. However, experiences that have been reported by participants for the most part suggest a compartmentalized faith, which apart from certain behaviors, seems to make very little difference in the everyday lives of participants. In other words, church involvement is inviting our children to be “better behaved and nicer,” but makes little difference to more holistic measures of health (with the significant exception of forgiveness). This suggests not only significant theological problems in the church, but also practical problems regarding ministry to children in the church.

## Discussion

Three core questions emerged from this qualitative study. The first issue raised is this: “*Why is a child*’*s physical life is not seen as an integral part of the Christian life?*” The second pertains to the question: “*Why is the fullness of life in Christ typically imagined as something that is limited to moral behaviors and church practices rather than extending into every area of our lives?*” The third was more positive: “*What glimpses does this study give us of the possibility of what church involvement can be in the lives of young people?*”

1. Why is a child’s physical life not seen as an integral part of the Christian life?

Our program of research is based on the theological assumption that the Christian life is not just about the saving of souls, but also about the whole human experience (with considerations of body, mind and spirit). A reality of being human is that we have bodies. We eat, we sleep, we play hard, run fast, become sick and are healed, and eventually, we grow old. This “embodiedness” is fundamental to our very being as humans and as Christians. To be human is to be temporally and spatially located within the settings of everyday life. Likewise, the Christian life is not lived in some distant, eschatological future, but in the fullness and complexity of life *here and now* as we anticipate in the present God’s ultimate re-creation of all things.

Our quantitative and qualitative findings then are cause for concern and suggest that most Canadian children who attend church understand their own physicality as having little to do with the Christian life. The one exception was the recurrent view that the Christian life does have something to do with the human body when connected with moral behaviors, such as smoking or drinking. These results suggest that Canadian children are missing out on some aspects of the good news of the Christian life: The gospel is not an escape from our bodily reality, but the healing and renewing of it.

The experiences reported by young people suggest a shallow understanding of the theology of the incarnation and resurrection that is central to our lives as God’s people. Indeed, the reported experiences reflected an understanding of human physicality and the body that emphasizes the spiritual over the physical. This in turn, thus suggests that the Canadian church continues to be influenced by a dualistic theology that puts a wedge between the spiritual and physical realms and which holds the one as ultimate to the other (Wright 2011). A natural implication of this theological error is that participants were unable to see the physicality of their bodies as a part of God’s story of redemption or as gifts to be celebrated. In short, we suggest that the participants in this study have been recipients of erroneous and/or shallow teaching (both explicit and implicit) in the church. They have been shaped and formed by this dualistic theological framework and this has resulted in their exclusion from the richness that they would potentially experience if they understood that rather than the physical realm being inferior to a moral/spiritual realm, the physical realm is precisely where God meets us in Jesus. Moreover, this physical realm is not only the location of the redemption story of which we are a part, but is included in and fundamental to God’s creative and redemptive action in the universe.

2. Why is the fullness of life imagined as something that is limited to moral behaviors and church practices rather than extending into every area of our lives?

Findings also suggest that the essential mission of the church may have become distorted by theologies that primarily offer a moral code to be followed rather than an invitation into the new life in Christ (Torrance 1996). This implies that in the church, there is a focus on teaching children how to behave rather than helping them to see the world and themselves in light of the dynamic and active presence of a sustaining, redeeming and creative God. It would be a distortion to suggest that the Christian life does not carry with it a particular understanding of morality, but this morality is a response—not a precursor—to the gracious actions of God on our behalf.

This focus on behavior has shaped many of the resources used in teaching children about the Christian life: our children learn very quickly that following Jesus is more about social conventions and moral behavior than it is about a relationship with our dynamic, loving and creative God. It is no great surprise, then, that although these findings demonstrate church involvement to be correlated with an increase in pro-social choices and a decrease in overt risk behaviors that are generally connected with morality, it does not appear to be correlated with the whole of life.

Theology ultimately shapes our practice. When we are working with a theology that has a tendency toward dualism, and that puts a primary emphasis on morality, we should not be surprised when participants tell us things like: “church is important to hockey because I am not mouthing off to my coach.” For this participant, the connection between a sport that she loves and his Christian faith does not appear to go deeper than her moral behavior. As a result she misses out on understanding that the joy of her bodily situation is an extravagant gift from God, one to be celebrated in her life of faith.

The “missed possibility” that much of the church is relinquishing is in substituting a narrow and dualistic version of the Christian life for the integrative, fullness of life that is promised in the Christian gospel. This promise of new life is not an invitation into some future, disembodied life and nor it is a call to a narrow, moralized life right now. Rather, the life Jesus offers to us in the gospel is an invitation into a *whole new way of being* in *this* world as God’s people and as citizens of God’s kingdom.

It is important to emphasize that we are not inferring that church involvement should lead to a trouble free or successful life. Indeed, the church operates within the confines of a broken world, and in its fullest sense, this promise of the fullness of life is an eschatological hope rather than a present reality. Moreover, the call to the Christian life is often in conflict with societal norms and values; conflict which eventually led to the crucifixion and death of Jesus. Jesus himself asserted that to follow him was to take up one’s cross daily. Inevitably, there are ways in which the Christian life will result in feelings of conflict and uncertainty as a person works to integrate their relationships in society at large with their relationships in the church.

3. What glimpses does this study give us of the possibility of what church involvement can be in the lives of young people?

Findings from this study should not all be viewed as negative, and indeed, the positive aspects of this story should be recognized and celebrated. Observations of reports of lower risk behaviors, higher pro-social behaviors, and the better sense of the overarching need for forgiveness and participation in acts of giving and receiving forgiveness reported by church-involved adolescents demonstrate that on some levels, the church is succeeding in positively impacting the lives of its young members. Numerous studies demonstrate the negative health trajectories of risk behaviors such as binge drinking (Miller et al. 2007), illicit drug use (Escobedo et al. 1997) and early initiation of sexual activity (Kandel et al. 1986). Pro-social behaviors such as helping and sharing also have strong connections to numerous positive health outcomes, including positive mental health (Schwartz et al. 2003) and greater longevity (Corporation for National and Community Service 2007). Participation in pro-social behaviors such as helping and sharing are in keeping with much traditional Christian teaching and practice, and are also strongly connected with positive measures of child health. Christian doctrine and formation appear to be important as a determinant of pro-social behavior.

The measure “feeling sorry for things I do” was initially difficult to interpret on the basis of our quantitative findings. While church-connected children did not report higher prevalence values with respect to the first three emotional health measures, they did report more heightened feelings of “feeling sorry for things I do.” Qualitative participants unanimously agreed that feelings of being “sorry for things I do” were positive and inherently connected to the concept of forgiveness. In turn, forgiveness is integrally connected to positive health outcomes. For example, current evidence suggest that: forgiveness is importance in the area of coping strategies (Worthington and Scherer 2004); forgiveness may impact health through reducing unforgiveness rather than creating positive emotional experiences (Harris and Thoresen 2005); forgiveness can affect both physical and mental health (Witvliet and McCullough 2007; Worthington 2005); and forgiveness promotes positive and pro-social emotions for victims (Witvliet et al. 2001); and offenders (Witvliet et al. 2002). One study demonstrated the role of forgiveness in replacing negative emotions and its relationship with aspects of physical health (Worthington et al. 2007). Many studies have confirmed the positive impact of forgiveness interventions in medical settings (for example, in areas of chronic pain (Rippentrop et al. 2005), cancer (Hansen 2002) and cardiovascular health (Worthington 2006). While all of these studies were conducted with adults, sufficient research exists to suggest strong relations between forgiveness and many aspects of health, and that it is reasonable to suspect we would see similar health benefits in populations of young people. This certainly can be viewed as good news in terms of the relationship between positive health and the experience of church-connected children.

Theologically, the emergence of forgiveness as such a positive theme in the qualitative study is deeply significant. A life characterized by forgiveness, both of God’s forgiveness toward us and of our participation in God’s forgiveness toward others, is central to life in Christ: not a moral code with sanctions attached, but as God’s way to life (Wright 2008). The experiences reported by our participants resonated with these theological ideas; they described at great length how the forgiveness they experience from God defines who they are, sets them apart within their peer groups and roots them in an experience of God that frees them to live within a framework that takes care of guilt, regret and anger through Jesus. The concept of forgiveness is central to who we are as God’s people. The children who participated in this study presented a unified view that forgiveness was a significant part of their church experience, of the way they live the Christian life, of what they have learned at home from their parents, and was an experience that impacted their lives beyond the church walls in every other sphere.

In terms of church ministry, findings surrounding forgiveness demonstrate that, for some determinants of health, the church is already moving beyond behavior in terms of the nurturing of children. This demonstrates that our ministry with children appears to be making a positive, tangible impact on the lives of children for some particular concepts. If the same emphasis that has been placed on forgiveness was also put on other areas of emotional well-being that are in keeping with the core message of life that Jesus teaches and embodies, we suggest that we would potentially observe similar results.

## Strengths and Limitations

Strengths and limitations of this qualitative study warrant comment. A major strength is the rich qualitative observations that emerged. Our data were textured, deep and nuanced, and observations were shared honestly and with generosity. Participants represented a range of ages, both sexes and a diversity of denominational, school and family experiences.

As the second component of a mixed-methods study, our study is unique and our findings and their interpretation benefit from two types of inquiry. Because the qualitative study was conducted following the quantitative, it was somewhat hypothesis-driven and focused on issues that had already been identified as potentially important in understanding relations between church involvement and health.

A further strength is that this study is based on questions with significant practical value. Observations were rooted in honest, real-life experiences. This provides concrete evidence to support both recognition of where the church is functioning well and suggestions as to where the church needs to change in terms of its ministry to and with children in the church.

The qualitative study was limited geographically to a single community in Ontario, Canada and to a limited number of denominations. Further, because the pastors of the respective churches recommended all participants, there was undoubtedly bias in the selection of children with the most positive attitudes for involvement. Findings from the interviews are necessarily not therefore expected to be generalizable to a full spectrum of young people.

This study was also based on one interview with each participant and thus does not take into account how their experiences might be reflected on a different day, what their experiences might be during a different season of life, or what their experiences might be after they have matured through natural development.

## Conclusion

This manuscript reports on the qualitative component of a mixed-methods study of the potential influence of church involvement on the health of young people. Collectively, findings raise important questions and concerns with respect to ministry to children within the Christian church, which pertain to the role of ministry in the health of children and especially the integrated experience of faith in their lives. While involvement in a church appears to offer some protection with respect to engagement in certain measured behaviors (particularly participation in risk and pro-social behaviors), one is left to wonder why this trend does not extend into other more far-reaching areas of life. There is potential for something better. These findings are sobering because the Christian life, and by extension of the church, has much more to offer our young people than what they seem to be receiving. The Christian life is about far more than behavior and extends into every part of our lives.
